# Giant Intrapulmonary Teratoma: A Rare Case

**DOI:** 10.1155/2011/298653

**Published:** 2011-09-25

**Authors:** Rayees Ahmad Dar, Majid Mushtaque, Sabiya Hamid Wani, Rayees Ahmed Malik

**Affiliations:** ^1^Department of General and Minimal Invasive Surgery, Sher-i-Kashmir Institute of Medical Sciences, Soura, Srinagar, Jammu and Kashmir 190011, India; ^2^Department of Cardiovascular and Thoracic Surgery, Sher-i-Kashmir Institute of Medical Sciences, Soura, Srinagar, Jammu and Kashmir 190011, India; ^3^Department of General and Minimal Invasive Surgery, Government Medical College, Srinagar, Jammu and Kashmir, India; ^4^Department of Pathology, Sher-i-Kashmir Institute of Medical Sciences, Soura, Srinagar, Jammu and Kashmir 190011, India

## Abstract

Teratomas are tumors composed of tissues derived from more than one germ cell line. Pulmonary teratomas are rare and commonly involve the upper lobe of the left lung. Criteria for pulmonary origin are the exclusion of a gonadal or other extragonadal primary site and origin entirely within the lung. We report a case of a giant pulmonary teratoma in a 2-year-old male child and review the relevant literature.

## 1. Introduction

Teratoma is a common tumor of the mediastinum but is rarely found in the lung [1]. Furthermore, a mature teratoma seldom metastasizes to the lung [2]. Mature teratomas are the most common histological type of germ cell tumors, followed by seminomas [3]. These lesions originate from the third pharyngeal pouch and may manifest with a variety of clinical and radiological features. Primary lung teratomas have rarely been reported since Mohr's description of this entity in 1839. Germ cell tumors are predominantly found in gonads, while the anterior mediastinum is the most common extragonadal site.

## 2. Case Presentation

**Figure 1:**
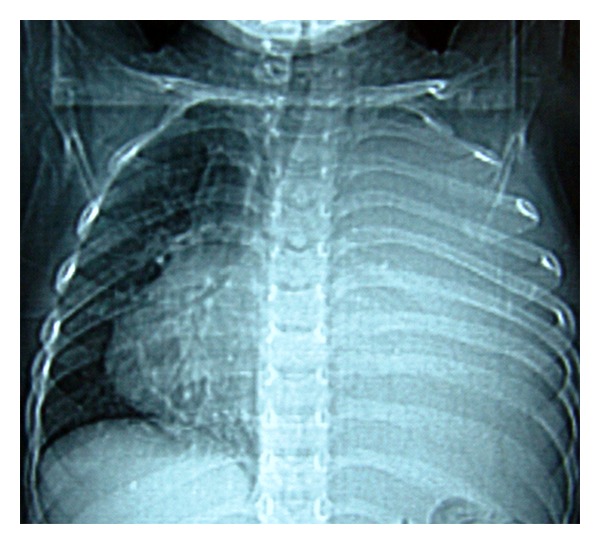
X-ray Chest PA view showing large opacity of entire left hemithorax with mediastinal shift.

**Figure 2:**
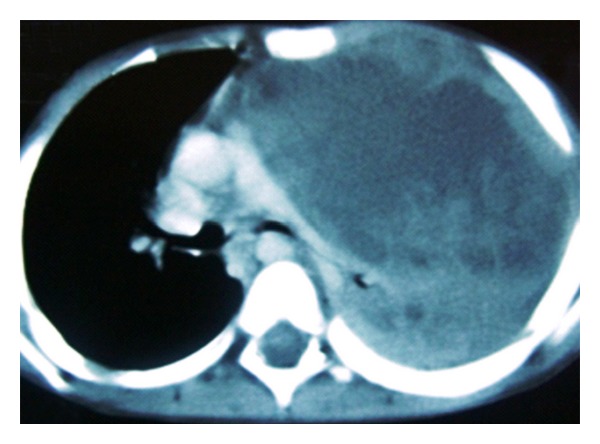
CT chest revealing a huge mass occupying the entire left hemithorax with mediastinal shift to opposite side (fat is not delineated nicely).

**Figure 3:**
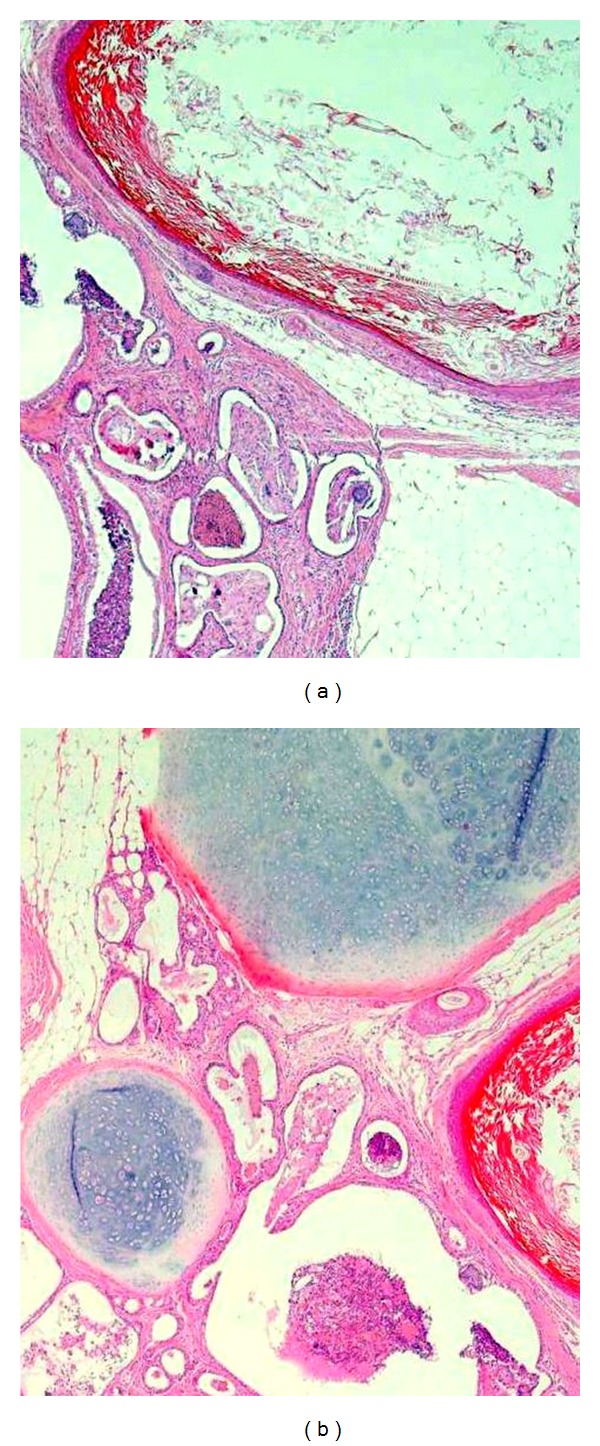
Photomicrographs of specimen of lung showing benign mature teratoma, containing stratified squamous and respiratory epithelium, mature adipose tissue, cartilage, and connective tissue.

A 2-year-old male child was referred from a peripheral hospital to our institute as a case of progressively aggravating dyspnea at rest, cough, intermittent fever, and recurrent chest infections from the last 5 months. The chest X-ray showed a large opacity of the entire left hemithorax (Figure 1). The child had been put on various antibiotic regimes, but the child's condition worsened day by day. On admission in our institute, the child was in acute distress. On chest auscultation, breath sounds on the left side were absent. There was no adenopathy or testicular mass. Computed Tomography chest was advised which revealed a huge mass occupying the entire left hemithorax with mediastinal shift to the opposite side (Figure 2). It was adherent to the left anterolateral subcostal pleura and the mediastinal pleura. The lesion showed heterogeneous density. No mediastinal lymphadenopathy, pleural effusion, or thickening was noted. Routine hematological tests and abdominal sonography were within normal limits. Mantoux test was negative. Tumor markers (*α*-fetoprotein and *β*-human chorionic gonadotropin) were both normal. Surgical management was first in the priority list of therapeutic options. A left-sided thoracotomy revealed a cystic tumor arising from the left lung. Many adhesions existed with the left pulmonary artery, the left main bronchus, the pericardium, the aorta, diaphragm, and parietal pleura. A combination of blunt and sharp dissection for the division was applied. A purse string suture permitted aspiration of sebaceous content via a small incision in the wall of the mass. As the size diminished, manipulation was facilitated. A pneumonectomy had to be carried out in order to remove the tumor mass. The child was shifted intubated to the Surgical Intensive Care unit in the immediate postoperative period. The child could not maintain oxygen saturation, and his condition deteriorated, and he expired on the second postoperative day. The histopathological examination revealed a benign mature teratoma involving the left lung, containing stratified squamous and respiratory epithelium, mature adipose tissue, cartilage, and connective tissue (Figure 3).

## 3. Discussion

The first case of pulmonary teratoma was reported by Mohr in 1839. Germ cell tumors occur typically in the second to fourth decades of life with a slight female preponderance. Our case is very rare keeping in view the age of the child. Mature teratomas are the most common histological type of germ cell tumors. Nearly one half of patients have no signs or symptoms when the mass is initially diagnosed. Symptoms commonly present are chest, back, or shoulder pain, dyspnea, cough, fever, pleural effusion, and bulging of the chest wall. Hemoptysis or expectoration of hair (trichoptysis) or sebum can rarely occur when communication between the tumor and the tracheobronchial tree develops. Trichoptysis is the most specific symptom [4]. Symptoms can also derive from the pressure exerted on the surrounding tissues (for example, superior vena cava syndrome). Intrapulmonary teratomas typically range from 2.8 to 3 cm in diameter and are cystic and multiloculated but may rarely be predominantly solid. In our case, it was exceptionally large tumor. Microscopically, mesodermal (bone, fat, and muscle), ectodermal (skin and hair), and endodermal (respiratory epithelium and gastrointestinal tract) elements are seen in varying proportions. Mature elements often take the form of squamous-lined cysts. Thymic or pancreatic elements may be seen in mature teratomas. Malignant pulmonary teratomas present as sarcoma or carcinoma with the presence of immature elements like neural tissue. In our case, tumor was composed of stratified squamous and respiratory epithelium, mature adipose tissue, cartilage, and connective tissue.

Diagnostic assessment is performed with classical X-rays, followed by Computed Tomography. Computed Tomography accurately estimates the density of all elements. Magnetic Resonance Imaging is valuable in detecting the anatomic relation to mediastinal and hilar structures, such as vessels and airways. Surgical resection is the treatment of choice; and radical extirpation leads to a long recurrence-free survival [5]. Since, in our case, tumor was involving the entire left lung, pneumonectomy was performed. Also taking into consideration the age and the very sick condition of the child preoperatively, his condition deteriorated in the immediate postoperative period and he did not survive.

## 4. Conclusion

Intrapulmonary teratomas are rare tumors. They originate from the third pharyngeal pouch and present as cystic lesions in the majority of cases. A giant teratoma involving the entire left lung, as seen in our patient, is noteworthy and has not been reported. Patients present with chest pain, cough, hemoptysis, and trichoptysis. Complete resection is the adequate treatment for patients with a good long -term prognosis.
